# Fostering engagement using a wearable for self-tracking assisted psychotherapy with refugees diagnosed with complex PTSD: a feasibility pilot study

**DOI:** 10.3389/fpsyt.2025.1250552

**Published:** 2025-02-26

**Authors:** Lisa G. Riisager, Lotte Huniche, Jakob Eg Larsen, Thomas Blomseth Christiansen, Lotte Kring, Sabina Palic, Stine Bjerrum Moeller

**Affiliations:** ^1^ Department of Psychology, Faculty of Health Sciences, University of Southern Denmark, Odense, Denmark; ^2^ Mental Health Services in the Region of Southern Denmark, Clinic of Trauma- and Torture Survivors, Vejle, Denmark; ^3^ Department of Applied Mathematics and Computer Science, Technical University of Denmark, Kongens Lyngby, Denmark; ^4^ Konsulent Blomseth, Hjørring, Denmark; ^5^ Danish Institute against Torture - DIGNITY, National Rehabilitation Clinic, Copenhagen, Denmark; ^6^ Department for Treatment of Borderline Personality Disorder and Self-Harm, Psychiatric Centre Glostrup, Mental Health Services in the Capitol Region of Denmark, Copenhagen, Denmark

**Keywords:** one button tracker, wearable technologies, complex PTSD (CPTSD), refugee, personalized psychotherapy, engagement, personal science, self-tracking

## Abstract

**Background:**

To address the unique challenges faced by refugees diagnosed with complex post-traumatic stress disorder (CPTSD), psychotherapy needs to be personalized. The integration of self-tracking instruments into therapy offers a promising approach to personalizing treatment. This feasibility pilot study develops and explores a preliminary self-tracking assisted treatment concept using a wearable self-tracking instrument called the One Button Tracker (OBT). The OBT is a single-purpose self-tracking instrument, designed to track subjectively experienced phenomena.

**Methods:**

The feasibility pilot study adopted a participatory action research design, involving close collaboration between two therapists, two refugees diagnosed with CPTSD, and a research team. Quantitative data was collected from the OBT and qualitative data consisted of semi-structured post-treatment interviews and session logbooks. Reflexive thematic analysis was used for the interpretation of interview data. Quantitative data was used descriptively.

**Results:**

The integration of OBT into psychotherapy with refugees was found to be feasible, marked by consistent high engagement as seen in the self-tracking data. Five themes were generated from the interview analyses, across two contexts: therapy sessions (navigating between precision and alliance with the OBT, and data usefulness in therapy) and daily life (paradox of awareness, OBT as a sign of treatment, and following the Doctor’s orders).

**Conclusion:**

This feasibility pilot study illustrates the feasibility and therapeutic potential for integrating the OBT into psychotherapy for refugees with CPTSD to enhance engagement and personalization. The findings emphasize the necessity of an adaptive, personalized approach, vigilance regarding potential risks, and consideration of cultural factors. Further research is needed to refine this novel therapeutic approach.

## Introduction

As of 2022, the global refugee population exceeded 26 million individuals, with a staggering increase of over 5 million in a single year particularly due to the war in Ukraine (1). Refugees have often endured prolonged and severe traumatic experiences, including torture and warfare. The effects of these experiences persist even after resettlement, increasing their likelihood of suffering from PTSD by tenfold compared to the general population (2). Post-migration stressors, such as isolation, economic instability, language barriers, social stigma, and unmet expectations, continue to impact their mental health (3–5). Because refugee resettlement also brings about additional challenges including discrimination, concerns for left-behind family members, and acculturation struggles, the impact of daily stressors continues to affect mental health (6).

Complex PTSD (CPTSD), a newly recognized diagnostic category in the International Classification of Diseases 11^th^ Revision (ICD-11) by the World Health Organization (WHO) can be reliably differentiated from PTSD (7). CPTSD includes PTSD symptoms alongside disturbances in self-organization, such as affect regulation challenges, alterations in self-perception, and difficulties in interpersonal relationships (8, 9). A systematic review by Mellor et al. (10) on the prevalence of CPTSD among refugees and forcibly displaced populations found rates ranging from 2% to 86%, with significantly higher rates observed in the Middle East compared to populations resettled in Western countries (5-33%).

Clinical guidelines have been careful in offering treatment recommendations for CPTSD, due to the limited data available to support evidence-based practices (11). While trauma-focused therapy is the primary recommended treatment for PTSD within the general population (12), its effectiveness for refugees remains unclear. Empirical and methodological challenges limit the evidence base for its application among refugees (13, 14). Refugees often experience trauma that is uniquely severe and prolonged, distinguishing them from other populations (5). Additionally, their mental health continues to be affected by post-migration stressors (6). Consequently, the complexity of symptoms in CPTSD may compromise the effectiveness of conventional manual-based trauma-focused therapy for refugees (15). Critics of this approach emphasize its limitations in addressing the ongoing psychological stressors refugees face in daily life (16, 17). As a result, there is a need for alternative treatment models specifically tailored to refugees diagnosed with CPTSD.

The unique challenges that refugees face necessitate cultural adaptations to Western psychotherapy models (14, 18). Yet, traditional psychotherapy often encounters constraints, such as language barriers and cultural differences (19). Even when interpreters are used, treatment outcomes are modest (20). Investigating the acceptability of trauma-focused Cognitive Behavioral Therapy (TF-CBT) among refugees, Vincent et al. (4) reported that additional challenges like fear of repatriation and potential retraumatization during trauma-focused therapy may impede patient engagement. Consequently, the need for a more personalized, culturally adaptive treatment approach is urgent.

In response to this, wearable single-purpose self-tracking instruments can potentially address the challenges of treating severe CPTSD psychopathology. Rooted in the personal science paradigm, self-tracking is a way of collecting data using empirical methods to answer personal questions (21). The premise is to ‘gain self-knowledge through numbers’ often with a focus on health-related issues (21–23). Using self-tracking instruments in clinical practice can serve as a potent tool to personalize treatment, as they can capture real-time data on patient experiences, behaviors, and emotions (24). By providing a language of data, they help elucidate the unique health-related phenomena that patients encounter. Consequently, these instruments may be instrumental in overcoming the hurdles to treatment, as they facilitate patient engagement and promote a personalized, culturally sensitive approach to therapy.

A recent review on digital mental health interventions highlighted their potential to support mental health but also identified challenges with engagement and a lack of personalization, both of which negatively affected outcomes (25). Traditional multi-step processes involving Ecological Momentary Assessment (EMA) wearables can impose a substantial burden on participants, as their time-consuming and demanding nature can further hinder engagement (26). These challenges are particularly pronounced among refugee populations, who frequently face higher therapy discontinuation rates due to extensive trauma histories (27). Developing less burdensome technology is therefore essential to make self-tracking technology feasible in real-world clinical settings (28). While self-tracking wearables promise to revolutionize psychotherapy by enhancing personalization and patient engagement, their integration into psychotherapy with refugees diagnosed with CPTSD remains unexplored.

### Rationale and aim

In a feasibility pilot study, we developed and explored a preliminary self-tracking assisted treatment concept using a novel wearable self-tracking instrument called the One Button Tracker (OBT). We aimed to explore the feasibility of using the instrument in psychotherapy with refugees diagnosed with CPTSD and to understand how self-tracking data could be integrated into the treatment approach.

We posed two research questions:

RQ1: How does the OBT mediate patient engagement in psychotherapeutic treatment?
RQ2: How does personalized self-tracking data become a part of the psychotherapeutic process?

By investigating these research questions, we aimed to gain a deeper understanding of patients’ and therapists’ experiences when integrating a self-tracking instrument into psychotherapy and identify the essential elements that should be considered. Significantly, our study represents the first of its kind to explore the integration of a single-purpose self-tracking instrument into intercultural psychotherapy for refugees, marking an innovative approach to addressing the unique challenges faced by this population.

## Method

**Figure 1:**
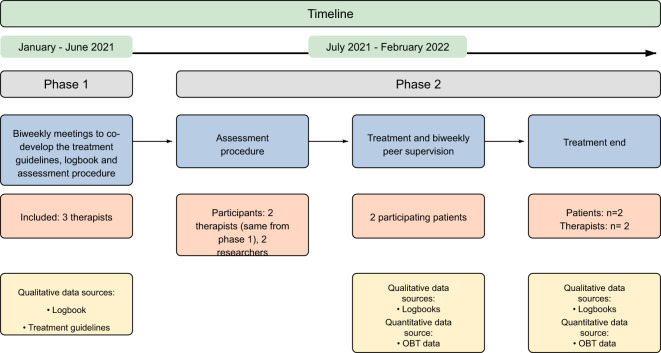
Flowchart of the PAR process and data sources.

The feasibility pilot study used a Participatory Action Research design (PAR, as shown in **Figure 1**), fostering collaboration between therapists and researchers in the iterative development of the treatment concept (29). PAR is a dynamic research methodology characterized by a continuous exchange between action and reflection, with methods and action modes evolving. This ongoing reflexivity allows for the continuous refinement and adaptation of the understanding and changes emerging through PAR, meeting the evolving needs of the group (30). The integration and specific clinical use of the OBT was explored in two ways during the research process: (1) iterative evaluations during peer supervision meetings, and (2) semi-structured interviews post-treatment that allowed both patients and therapists to evaluate the co-developed treatment concept. The insights and experiences gained from this feasibility pilot study will inform the design of a larger, subsequent study. The study was evaluated by The Regional Committees on Health Research Ethics for Southern Denmark (project-ID: S-20210019 CSF).

### Setting

The study took place at the Clinic for Trauma and Torture Survivors (CTTS) within the Mental Health Services, Southern Region of Denmark. This clinic specializes in treating refugees and veterans diagnosed with PTSD and CPTSD after experiencing war, political persecution, and/or torture. The treatment approach at CTTS is multidisciplinary, involving close collaboration between psychologists, psychiatrists, physiotherapists, nurses, and social workers to determine the most appropriate treatment approach for each patient based on clinical assessments of their psychopathology and level of functioning. Psychotherapy is conducted by psychologists who receive monthly supervision, primarily based on a CBT and narrative exposure therapy approach, as well as monthly interdisciplinary supervision. Treatment duration varies depending on the severity of psychopathology as well as social and somatic challenges. When requiring interpretation, interpreters are virtually present in sessions via a software application running on an iPad tablet.

### Procedure and participant recruitment

This study was executed in two distinct phases. During the initial phase, a collaborative research alliance was established, compromising first author LGR and supervisor SBM, along with the participating therapists, who also served as co-researchers. Both LGR and SBM have a background as clinical psychologists, with LGR further specializing in qualitative research and SBM demonstrating substantial experience in psychotherapy research. This collective engaged in biweekly iterative discussions for ninety minutes over five months, aiming to generate a preliminary self-tracking assisted psychotherapy concept (see **Figure 1** for a flowchart of the PAR process and data sources). Initially, three therapists participated in the study, but one resigned from her position at the clinic as phase 2 was starting and withdrew from the study. The participating co-researchers were two psychologists, one with extensive experience in treating trauma but with limited experience in working with refugees, and the other with specialized experience in conducting psychotherapy with refugees. Both therapists primarily used 2nd and 3rd wave CBT approaches.

The second phase involved patient recruitment and treatment. Patients suspected of CPTSD during intake clinical interviews at CTTS were referred to the study. Eligibility criteria included: (1) being referred for psychiatric treatment at CTTS, (2) having a status as a refugee, (3) age 18 years or older, (4) having a CPTSD diagnosis according to The International Trauma Interview (ITI)^1^. A Danish version of the ITI was used in this study, which was translated from English by Sofie Folke, alongside a team of researchers and clinicians from the Danish Veteran Centre, building upon an earlier version developed by the Competence Centre for Transcultural Psychiatry, Mental Health Centre Ballerup, Denmark (31). The Danish version of the ITI has undergone reverse translation from an independent professional translator, which was subsequently reviewed and endorsed by the original ITI author group, to validate its consistency with the intended diagnostic framework. Interpreters participated in the therapy sessions through an iPad application. Participating patients provided oral and written informed consent in their native language through an interpreter. Three patients were initially recruited, but one patient withdrew shortly after beginning the treatment. Unfortunately, it was not possible to arrange a follow-up interview with the patient who withdrew. The remaining two patients both needed interpreter-mediated therapy (see **Table 1** for patient characteristics).

**Table 1:** Patient characteristics.

Patient characteristics		
	Patient 1	Patient 2
Gender	Male	Male
Age	46	53
Country of origin	Syria	Iraq
Married	Yes	Yes
Education	No professional education	Shorter higher education
Employment	Unemployed	On sick leave

Data collection spanned from July 2021 to February 2022. After participant recruitment, the biweekly meetings transitioned into peer supervision sessions, aiming to provide case supervision, collect ongoing qualitative data regarding treatment experiences, and continuously refine the use and integration of the OBT in psychotherapy sessions.

### Treatment concept

The preliminary self-tracking assisted treatment concept developed for this pilot study incorporated three core elements: The One Button Tracker, Guidelines for integrating the use of OBT in therapy, and a logbook. The treatment concept was iteratively designed through collaboration between the research team and participating therapists during phase 1 and was continually refined throughout the treatment periods based on session experiences. This co-creation process tailored the psychotherapeutic concept to meet both therapists’ and patients’ needs and preferences, enhancing its relevance in real-world clinical settings.

#### The one button tracker

**Figure 2:**
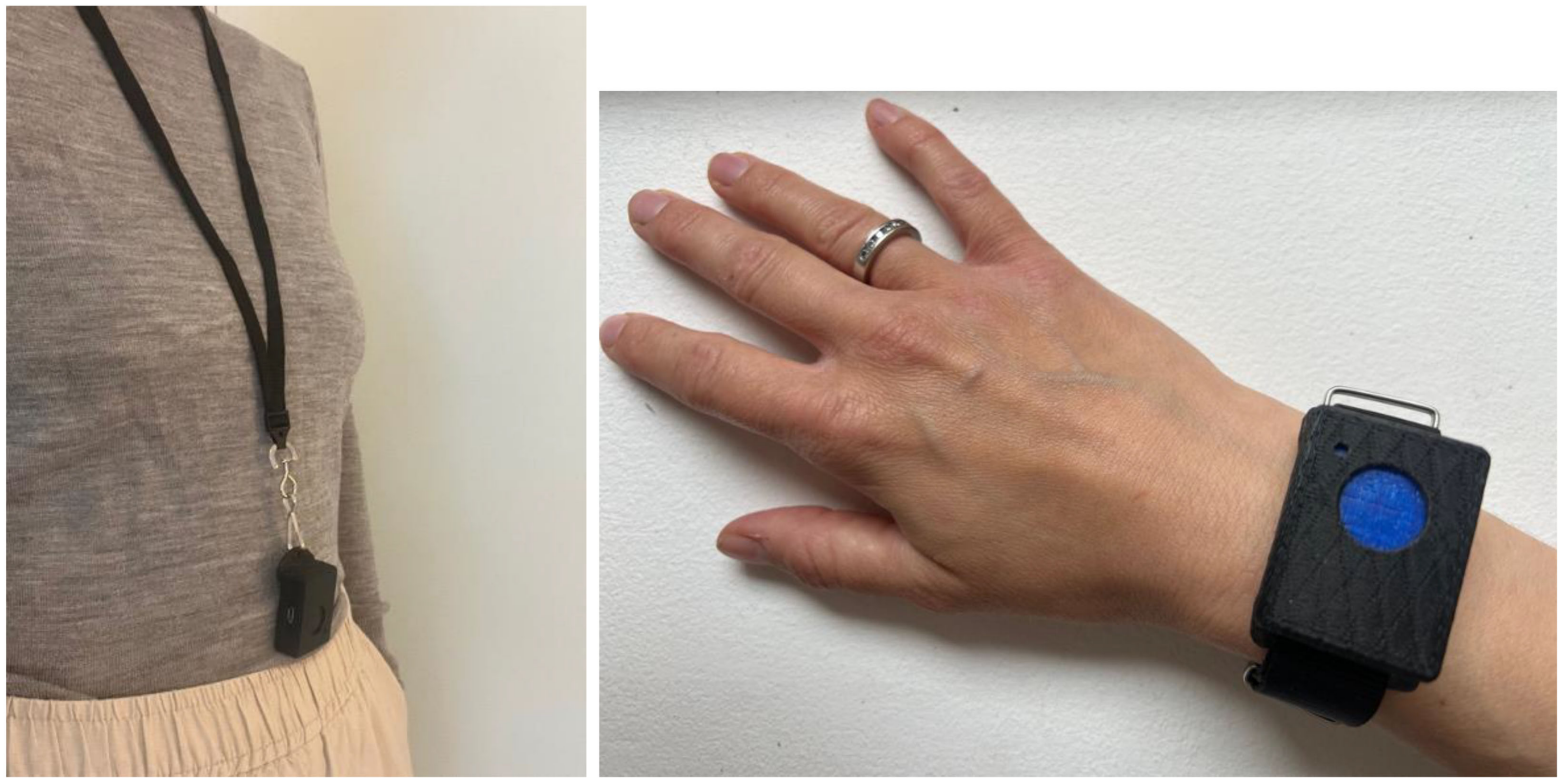
Examples of the One Button Tracker (OBT) worn around the neck and on the wrist.

The OBT is a small, single-purpose, wearable self-tracking instrument. We intentionally use the term ‘instrument’ as opposed to ‘device’ to emphasize its specific function in collecting data, akin to a measurement tool. The term ‘device’ carries broader connotations that might not capture the precise function of the OBT. The instrument is designed to register observations of a subjectively experienced phenomenon quickly and discreetly with a single button press, eliminating the need for visual reference. Coauthors JEL and TBC invented the instrument and pilot-tested it with individuals with PTSD and CPTSD (32–34) and among employees of an academic medical center (35). The prototype used in the study was 41×31×12.5 mm and could be worn around the neck, in a pocket, or as a wristband (see **Figure 2**). Observations were registered by pressing the button. The instrument provided vibrotactile feedback for the duration of the button press, signaling that an observation had been recorded and stored. Data was transferred via a USB cable to a computer, and a web-based data visualization tool allowed analysis of the temporal occurrences and distribution of the phenomenon.

#### Self-tracking assisted psychotherapy

Inspired by the practices of the Quantified Self community (21), the therapy incorporated the OBT framed as a personal diary tool for the patients, enabling them to record a personally significant phenomenon in their daily lives with a press of a button. At the core of this treatment approach lies the *target phenomenon*. This is a subjectively defined distinct experience, whether it’s a triggering stimulus (e.g., heart palpitations, shortness of breath, conflicts), a symptom (e.g., flashbacks), or a challenging behavior (e.g., angry outbursts, isolation). When the OBT is introduced into psychotherapy, the patient and the therapist collaboratively identify the target phenomenon, ensuring high motivation in self-tracking its occurrence. As the therapy progresses, the target phenomenon is continually documented and updated, with adjustments being informed by ongoing data analysis. This method ensures that the therapeutic process remains dynamically responsive to the patient’s changing circumstances and therapeutic needs.

To anticipate the data that the OBT would capture on the target phenomenon, a *hypothesis list* was developed, outlining the patient’s expectations (e.g. how often the target phenomenon would occur, at what time of day or on certain days, under which circumstances, etc.). This encouraged patient engagement and helped identify their assumptions about the target phenomenon.

The process by which the patient records their target phenomenon was defined in the *observation protocol.* This included an agreement on how and when the patient decided to press the OBT button. The initial recommendation was a one-press protocol, where a single button press registers the occurrence of a single target phenomenon. If necessary, this protocol could be expanded to a two-press observation protocol to record a subsequent target phenomenon or intervention. To ensure that the self-tracking activity was clinically focused and manageable for the patients, an *evaluation of the observation protocol* was conducted. This assessed the tracking period between sessions, the patient’s tracking experience, and their ability to distinguish the target phenomenon from other phenomena of their daily living during the tracking period.

The therapy’s unique feature lies in its *in-situ* and in-the-moment data collection via the OBT, offering distinctive insights into the interconnections between the patient’s phenomena and daily challenges. Encouragement to utilize the OBT was confined strictly to therapy sessions, with no dedicated efforts extended to prompt its usage beyond therapy sessions.

**Figure 3:**
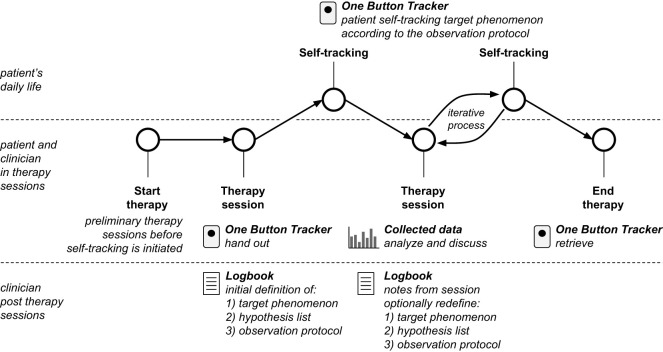
Flowchart of the therapeutic process involved in the self-tracking assisted treatment concept where a patient uses the One Button Tracker in between therapy sessions to self-track a selected target phenomenon agreed upon during a therapy session. Clinicians keep a logbook including 1) target phenomenon, 2) hypothesis list, 3) observation protocol, and notes from a therapy session. The process is iterative as analysis and discussion of data during therapy sessions may lead to redefinition.

The reciprocal process between ‘therapy session’ and ‘self-tracking’ in the flowchart shows the iterative nature of the treatment concept (**Figure 3**). After the initial definition in therapy sessions and data collection in the self-tracking period, the data is analyzed during therapy sessions. This iterative process informs adjustments to the target phenomenon and tailors relevant interventions, ensuring that the therapy remains personalized to the patient’s evolving needs and experiences.

#### The logbook

The logbook served as a tool for therapists for session documentation and reflection, and it incorporated the four main components of the treatment: (1) documenting the target phenomenon, (2) a hypothesis list outlining what the patient expected to observe in the data, (3) the observation protocol, and (4) an evaluation of the observation protocol.

### Data sources and data collection

The analysis is based on two primary sources of data: (1) OBT data, recording self-tracking from patients’ daily activities consisting of 98 days of OBT data from Patient 1 and 106 days of OBT data from Patient 2 and (2) semi-structured interviews with patients and therapists upon treatment completion. Complementing the semi-structured interviews, logbooks provided details on each treatment session. **Table 2** provides an overview of the data corpus.

**Table 2:** Overview of the two patients’ OBT data.

Participant	Tracking started (session)	Duration (days)	Number of observations	Daily avg. observations	Days with observations
1	3	98	716	7.3	98 (100%)
2	4	106	443	4.2	95 (90%)

The data from the OBT provided quantitative information on the occurrence of the target phenomenon in the patient’s daily life as well as the patient’s overall engagement in using the OBT as adjunctive to therapy.

The semi-structured interviews elicited perspectives and experiences from patients and therapists regarding their use of the OBT. The interviews, guided by a semi-structured interview guide for each participant group, were continuously developed throughout the treatment period. Interviews with patients aimed primarily at understanding their experiences of using the OBT in their treatment. Adopting a narrative approach, these interviews encouraged patients to share their life experiences and weave the meaning of their treatment into their narratives (36). Conversely, interviews with therapists aimed to explore their experiences and perspectives on integrating the OBT into their psychotherapy practice. Beyond capturing their experiences with the OBT, these interviews provided insight into psychotherapeutic processes, focusing on how the inclusion of patients’ self-tracking data affected treatment sessions and the overall therapeutic journey. This investigation illuminated the impact and potential of the OBT in therapeutic settings from both patient and therapist viewpoints.

Author LK conducted interviews with two therapists at CTTS and with the two patients, one at CTTS and one at the patient’s home. Given the language barriers, both patient interviews were aided by an interpreter present through an application on an iPad. All interviews lasted between 45-60 minutes and were audio recorded and transcribed verbatim.

### Data analysis

The quantitative OBT data was the starting point for the analysis which involved several steps. Initially, the data was plotted in various visualizations to discern patterns and trends in patients’ observations. These included a calendar-style plot and histograms illustrating the number of daily observations, as well as the distribution of observations by weekdays and time of day. Moreover, summary statistics such as average daily observations and the ratio of days with observations relative to the total tracking period were calculated to quantify patient engagement. These visualizations were also utilized in the therapy sessions for analysis with the patient. OBT data offered insights into patients’ engagement with both the instrument and with therapy, thereby serving as a preliminary indicator of feasibility. This quantitative analysis informed the subsequent exploration of the qualitative data, which provided in-depth insights into the experiences and patterns associated with using the OBT in therapy.

The qualitative data set, consisting of semi-structured interviews with patients and therapists, was supplemented by notes from logbooks. The semi-structured interview data was analyzed using the reflexive thematic analysis (TA) approach following Braun & Clarke’s six phases used iteratively: Familiarization, data coding, initial theme generation, reviewing themes, defining and naming themes, and writing up the findings (37, 38). Analysis was performed by the first author LGR under co-authors’ supervision. The flexibility of the TA method allowed LGR to inductively use an experiential approach, developing codes and themes iteratively within the dataset. As a result, the analysis evolved from primarily semantic to include a more latent orientation. Moreover, the approach’s theoretical flexibility allowed the analysis to be informed by the postphenomenological philosophy of technology, focusing on the OBT’s role in mediating patients’ treatment engagement and how the self-tracking data becomes a part of their treatment.

The data analysis process started with LGR and LK (an experienced qualitative researcher and anthropologist) familiarizing themselves with the dataset and discussing potential codes. Interviews were coded separately using both semantic and latent codes. The coding process was dynamic, with codes reviewed continuously for precision in their labeling. Codes were discussed with co-authors continuously throughout the coding process. After examining the codes and the coded dataset, LGR generated themes for each dataset based on the latent meanings of the codes. The NVivo 12 software program assisted the coding and analysis process, enabling first author LGR to share and discuss codes and potential themes with co-authors. Initial themes linked to the research question were generated through clusters of inductive codes (37). To ensure the themes reflected the raw data, LGR continuously revisited the data corpus during the theme development and finalization stages of the analysis. Logbook data was used to inform the interview analysis but not analyzed.

## Findings

The findings are presented in two sections: the first section includes findings on feasibility and engagement as shown by OBT data, while the second section presents findings based on semi-structured interviews.

### Feasibility and engagement in self-tracking: OBT data

The feasibility of the self-tracking assisted psychotherapy concept and the engagement level of the two participating patients is demonstrated by the data gathered by the patients using the OBT instrument. The patients started using the OBT at sessions 3 and 4, respectively, and continued tracking for 98 and 106 days. An overview of the self-tracking data collected by the two patients can be seen in **Table 2**.

Patient 1 consistently made observations on all 98 days the instrument was used as a part of his treatment, while patient 2 made observations on 95 of the 106 days (90%) it was deployed. The 11 days (10%) without observations for patient 2 were due to the OBT instrument running out of power. The two patients recorded 716 and 443 observations in total, which corresponds to an average of 7.3 and 4.2 observations made per day.

**Figure 4:**
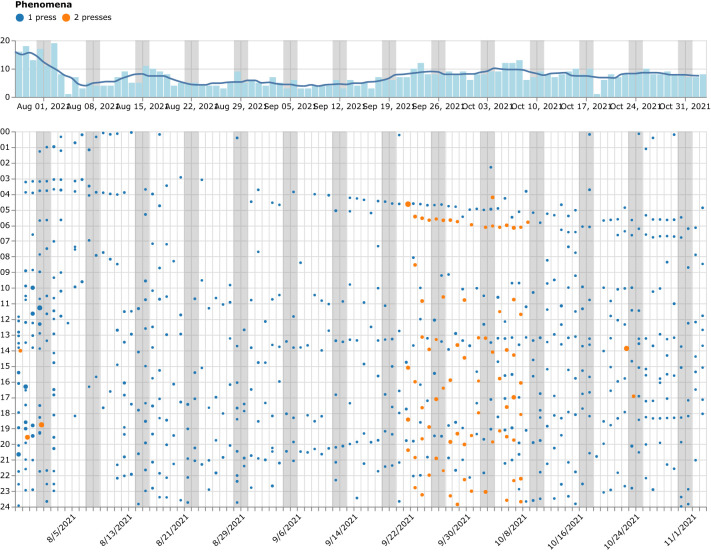
An overview of Patient 1’s observations in a calendar-like visualization during the treatment period with days on the x-axis (weekends marked light gray) and the hour of the day on the y-axis. Each dot represents an observation made with the instrument. The image shown is a screenshot from the web-based data visualization tool that was used during therapy sessions.

**Figure 5:**
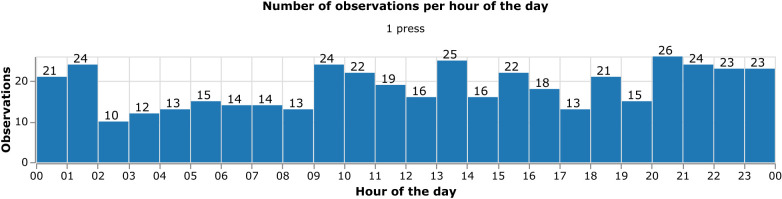
Patient 2’s total number of observations distributed by hours of the day.

**Figure 6:**
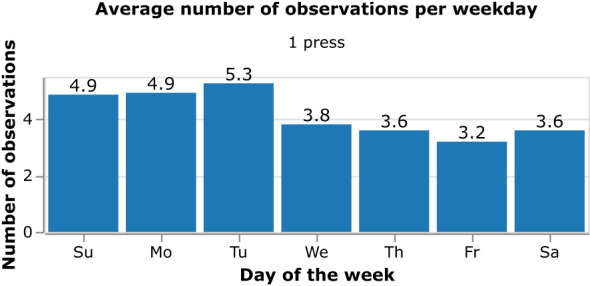
Patient 2’s average number of observations per day distributed across the days of the week.

During therapy sessions, the patient and therapist used a purpose-built, web-based data visualization tool to view and discuss the collected data. **Figures 4**–**6** in the following subsections provide screenshots from the web-based tool that was used by therapists and patients. The visualizations display all patient-collected data acquired using the OBT throughout treatment, reflecting the visualizations as they appeared to patients and therapists on the final day.

**Figure 4** provides a condensed calendar visualization, showcasing all observations made during the 98-day self-tracking period of Patient 1, thereby illustrating the high level of patient engagement. Each dot represents an observation of the target phenomenon made by the patient by pressing the button on the OBT instrument. Throughout the data collection period, the observation protocol underwent several modifications. However, most of the time, the patient observed a single target phenomenon at a time and registered it with a single button press (marked with blue dots). The observation protocol was also expanded for three weeks, starting around the midpoint of the data collection period, as denoted by the presence of orange dots (see **Figure 4**). During this time, two target phenomena were monitored, with one and two button presses assigned to each. The bar chart on the top of the calendar provides a count of observations made per day and the line provides a one-week moving average of the number of observations. This enables the patient and therapist to see the longer-term trend of the number of observations per day.

The high level of patient engagement and adherence to the observation protocol is indicated by the volume of observations made by both of the patients. **Figure 4** also demonstrates the high level of consistency in tracking as there are observations made on all days. An average of 7.3 observations were made per day by Patient 1 when counting both phenomena.

**Figure 5** shows the distribution of the number of observations made on the hours of the day for Patient 2. It makes clear that the observations of the target phenomenon are distributed across all hours of the day and demonstrate a high level of engagement in self-tracking and the use of the OBT instrument during the full 24-hour day.

**Figure 6** shows the distribution of the average number of observations made per day by Patient 2 and indicates the consistency in observations made for the target phenomenon across all days of the week, with a slightly higher average number of observations made on Sundays, Mondays and Tuesdays.

### The OBT as a part of psychotherapeutic treatment: Interview data

The OBT was used in two distinct contexts: therapy sessions and daily life. Most of the data from therapy sessions was generated by therapists, while patients generated data from daily life. Two themes were generated from the context of the therapy sessions and three from the context of daily life (see **Table 3** for a summary).

**Table 3:** Summary table of themes generated from the interview data.

Context	Themes
Therapy sessions	1. Navigating between precision and alliance with the OBT
	2. Balancing the use of OBT data in therapy sessions
Daily life	1. The effect of awareness
	2. OBT as a sign of treatment
	3. Following the Doctor’s order

### Context: therapy sessions

#### Theme: navigating between precision and alliance with the OBT

Integrating the OBT into therapy revealed intricate dynamics concerning therapeutic precision and alliance maintenance. Although therapists recognized the innovative potential of the OBT to enhance communication through data, they also reported challenges.

One challenge was defining a clear target phenomenon, especially when working through an interpreter. While this issue is not unique to the participants in this study, using the OBT may have made it more apparent compared to traditional therapy. One patient’s therapeutic process was marked by challenges in defining a shared, precise target phenomenon and tracking changes between sessions. Therapist 1, who had limited experience treating refugees, described the process as “saying the same thing over and over again” and “not the precision I had hoped for” (therapist 1) highlighting the ongoing difficulty in achieving clarity. The following excerpt from Therapist 1 illustrates the complexity of using the OBT to establish precision in tracking a specific target phenomenon:

*“We need to know the precise connections between “when I feel this, I press the button and then what happens?” Instead I got a long explanation that he … he explained to me that it was difficult for him during the war. “Okay”. (…) It was far from the question: “Did you press the button due to experiencing pain or an intrusion? “He … he didn’t really distinguish.”* (Therapist 1).

Despite the patient’s engagement with the OBT throughout the treatment process, therapist 1 encountered frustration. The root of this issue stemmed from the uncertainty surrounding the patient’s motivation for using the OBT, compounded by the inaccurate definition of the target phenomenon noted in the OBT data. This ambiguity posed challenges for the therapist in guiding the therapy toward the treatment plan’s objectives. The therapist’s logbook entries substantiate this difficulty, as they reflect the necessity to alter the target phenomenon five times during the treatment period.

However, the therapists’ experiences in achieving precision for the target phenomenon during their sessions varied significantly. Therapist 1 struggled to maintain precision throughout the treatment, while therapist 2 who had extensive experience in treating refugees, quickly established a clear target phenomenon with the patient. This enabled a more meaningful data causality analysis during therapy. Therapist 2’s success highlighted the importance of a shared understanding between the therapist and the patient regarding the rationale and definition of the target phenomenon. It also highlighted the critical role of the therapist’s familiarity with the refugee population and communication skills in guiding treatment, despite initial uncertainty about patients’ motivations. This was evident in Therapist 2’s reflections:

*“ (…) I quickly got a sense of how often it actually occurred to him at home. Usually, they tell us that it happens all the time, and then you have to guess how often that would be. Here, we could quickly analyze what happened when it occurred to him at home.”* (Therapist 2).

The ease with which therapist 2 established a shared understanding and precise treatment target with patient 2 demonstrated the mediating role of the OBT data in facilitating communication between patient and therapist. This observation was further confirmed by logbook notes documenting the therapist’s sustained focus on the same target phenomenon across nine sessions. According to the therapists, using the OBT and the OBT data enhanced the analytical process in therapy by creating an increased focus on the process of identifying a precisely defined target phenomenon, thereby providing the patient and therapist with a clearer insight into its actual occurrence.

In addition to precision, maintaining the therapeutic alliance was another significant concern for the therapists. The introduction of the OBT required them to continually shift their focus between the patient, the conversation, and the OBT data. They expressed concern that this shift could disrupt the therapeutic alliance, as it challenged their usual therapeutic strategies, such as “listening”, “understanding,” and “creating a contact.” (Therapist 1). The situation often left the therapists caught between maintaining the working relationship with the patient and the logistical demands associated with using the OBT, leading to a sensation of working beyond their usual therapeutic capacity. As Therapist 2 stated:

*“I felt a bit of time pressure at the beginning (…). To present [the OBT] and start investigating what we should use it for, and make it completely clear, what are you going to go home and do, what is your hypothesis about what will happen. I mean (…) from the start I was a bit worried about whether it would be at the expense of just meeting [the patient] (…)”* (Therapist 2).

This scenario left Therapist 2 negotiating between maintaining the working relationship with the patient and meeting the demands associated with using the OBT in therapy. This tension caused a sensation of working beyond their usual therapeutic capacity and disrupted the usual conversational dynamics.

From the patients’ perspective, the OBT and the therapeutic relationship existed as distinct aspects within their therapeutic experience. While they report any overt negative impacts of the OBT data on their therapeutic relationships, their reflections reveal a nuanced interplay between the therapeutic relationship, their perception of the OBT, and its use. For instance, Patient 2 described the OBT and the therapeutic relationship as separate and non-overlapping entities:

*“I don’t want to talk about the instrument. It doesn’t help me, it can help others, but it doesn’t help me. But [name of his therapist], she of course helps me, because we sit alone and talk together and sort of soothe my problems. It gets better when I talk with her.”* (Patient 2).

This quote underscores the perceived lack of meaning of the OBT in Patient 2’s therapeutic process. Yet, despite expressing that the OBT ‘did not help him’, the patient consistently used the OBT throughout the treatment period, indicating a discrepancy between his verbal expression and actual behavior. This disconnect underscores the complex dynamics of integrating the OBT into therapy, highlighting the nuanced role of the OBT as an adjunct to the therapeutic process rather than a primary agent of therapeutic change. Patient 2’s experience reveals that while the OBT might not be perceived as directly helpful, the therapy, within which the OBT was integrated, was perceived positively. This insight was echoed in an anecdote shared by Therapist 2:

*“(…) when we have found a target phenomenon, we have to manage both to talk briefly about the status from last time and if there is anything new that is important. Then we had to load the OBT and look at the screen, in principle, we also had to manage to work therapeutically with something, and it was just difficult. And then I remember, maybe the 7th, 8th, 9th time, the patient said, there is something that is important for me to tell you. And then he says, I have never felt as safe with anyone as with you before, or something like that. Then I just remember thinking that I can be somewhat okay with this. But I felt that I had to use more energy on ensuring that we had a connection because there was also another focus.”* (Therapist 2).

In this quote, the patient acknowledges a strong therapeutic bond, allowing him to explore and express his emotions and experiences, despite the therapist’s perceived challenges in integrating the OBT and its data into the treatment. The patient’s reassurance regarding the therapeutic alliance indicates that the use of the OBT and its data did not negatively affect his relationship with the therapist. Instead, it suggests that the safety and effectiveness of the therapeutic relationship resided within the dynamic, empathetic interactions between the patient and therapist, rather than the adjunctive use of the OBT. In other words, the strength of the therapeutic alliance may act as a mediating factor that allows patients to engage with novel tools like the OBT, even when they may not directly attribute their therapeutic progress to these tools.

#### Theme 2: balancing the use of OBT data in therapy sessions

The interviews with therapists elicited a blend of interest and hesitation concerning moments of balancing the use of OBT data during therapy sessions. The therapists acknowledged the OBT as a useful instrument for gaining insights into the prevalence and patterns of the target phenomenon in the patient’s daily life. The OBT, in providing detailed data on patient well-being and daily activities, facilitated data-informed clinical decision-making - a process that often proves difficult with traditional methods. A substantial advantage of the OBT, as emphasized by one therapist (Therapist 2), was its ability to overcome language barriers frequently encountered with conventional homework in intercultural therapy settings. Therapist 2 noted:

*“Often, if they have homework where they have to write, I can’t understand what they’ve written. So, it has actually fallen out of my therapeutic practice, because then we spend a lot of time on it the next time, to the point where we could spend almost an entire session explaining what they’ve written in their diary. So, I really rarely use homework. Therefore, being able to easily collect some data that’s not language-dependent is very useful.”* (Therapist 2).

This sentiment underscores the utility of the OBT as an instrument for gathering language-independent data, avoiding inefficiencies and misunderstandings that traditional homework could induce, especially within intercultural therapy contexts. Such high-frequency data collection offered valuable insights into the patient’s daily well-being and contextualized their experiences in a way that conventional methods could not.

Despite these advantages, a discrepancy emerged between the therapists’ and patients’ perspectives on the interpretation and utility of OBT data. This divergence was aptly illustrated by an interaction between Therapist 1 and patient 1:

*“Yes, and that’s what I talked to him about. I said, “it looks like there’s a subtle decrease in your symptoms.” Which he didn’t agree on. Or … that he couldn’t confirm. (…) he only experienced feeling bad. And it wasn’t better or worse or anything.”* (Therapist 1).

The patient did not seem to emotionally engage with the visualized data or the therapist’s interpretation, focusing instead on his overarching sense of unease. This detachment raised questions about the relevance of the data analysis within the therapeutic process and highlighted the divergence in perspectives. Therapists were often intrigued by specific patterns and occurrences within the data, while patients tended to view the OBT data as a holistic reflection of their mental health status.

These contrasting views on the utility of the OBT data were observed among study participants. Therapists regarded this data as an instrument for discerning occurrences and behavioral patterns, aiming to drive therapeutic changes such as increasing awareness on triggers or introducing alternative coping strategies. Patients, however, saw the OBT data as serving dual purposes: Providing insights into their mental health and facilitating therapeutic discussions. For example, Patient 1 found the OBT data to be a catalyst for therapeutic discussions. While acknowledging that discussing his pain (his chosen target phenomenon) would not change his situation, the tangible self-tracking data provided a foundation for meaningful dialogue.

*“My situation will not change just by talking about it. Because as I said, my pain exists and it gets worse over time. Perhaps one thing which is easier is when we talk about my pain, my therapist can see how much pain I have.”* (Patient 1).

This quote shows how data visualization enabled his therapist to access a more immediate understanding of his mental health (39). The digitized representation of his mental health facilitated the therapist’s exploration and understanding of his well-being. Consequently, the therapist was confronted with the task of managing the use of the OBT data while maintaining a connection with the patient’s perspective through the OBT. This case underscores the multifaceted role of the OBT in the therapeutic process: serving simultaneously as an instrument for tracking a phenomenon for the therapist, and as a way of gaining insight and therapeutic dialogue with the patient.

Nevertheless, the therapists acknowledged that OBT data wasn’t always relevant or meaningful, particularly in times of crisis. This understanding underscores the necessity of striking a balance between data-driven insights and individual patient experiences in therapy:

*“(…) I would also have liked to introduce an intervention if the treatment proceeded differently, but I didn’t succeed as he started to feel worse (…)”* (Therapist 2).

This quote from Therapist 2 illustrates how the use of OBT data within therapeutic sessions can be conditioned upon the therapists’ clinical judgment regarding the patient’s mental state in a given session. As detailed in the logbook notes, therapists often modulated the use of OBT data based on their clinical impressions of the patient in the specific therapy session. For instance, if patients experienced sudden grief, crises related to their home country, or overwhelming somatic pain, therapists would forgo the use of OBT data in that therapy session. While data analysis was a primary focus during initial sessions, as treatment progressed, therapists found it necessary to adapt their use of the OBT and the OBT data to accommodate the dynamics of the patient’s conditions. However, it is noteworthy that decisions to deviate from the use of the OBT and OBT data during certain sessions were not always explicitly communicated to the patient, despite a presumed agreement about its role in the treatment process. During a session, Therapist 2 made a therapeutic decision without consulting the patient, stating:

*“There was also a time when I didn’t ask him at all if he was willing to work with the tracker in the session. Instead, I made a therapeutic decision to focus on helping him regain his composure as a person because he was feeling overwhelmed by recent events.”* (Therapist 2).

Therapist 2, based on her judgment, deemed this therapeutic decision suitable for this specific patient. This decision was informed by the perceived alignment with the patient’s cultural expectations of the therapist-patient relationship, potentially alleviating him from the stresses associated with shared decision-making.

Substantiating the need to balance the use of OBT data in therapy sessions, was how Patient 2 found the visualization of his observations to be emotionally unsettling:

*“I felt stressed when I saw how many times I’ve pressed the button and how many times I’ve remembered what happened. It was actually unpleasant.”* (Patient 2).

For this patient, the self-tracking data confronted him with his target phenomenon, evoking feelings of vulnerability. His experience suggests that the OBT tracking activity can create an exposure effect, particularly for patients who tend to avoid distressing thoughts and emotions. Logbook notes show that this unintended exposure situation was unknown to the therapist and therefore went unaddressed.

### Context: daily life

#### Theme 1: the effect of awareness

Using the OBT in their daily lives seemed to increase the patient’s awareness of their respective target phenomenon, subsequently leading to various degrees of emotional distress. Both patients reported an increased awareness during the self-tracking process, with Patient 1 considering the experience as uncomfortable but manageable. Patient 2, however, experienced significant emotional distress from the intensified focus on his traumatic experiences, which he evaluated negatively. The tension in his account exemplifies that exposure to distressing thoughts can enable therapeutic attention, despite its potential discomfort:

*“I remember many things. When I press the button, I start to remember more. When I press again, I remember even more. The same thing happens, a war between me and the instrument. Every time I press the button on the instrument, even more happens. Just like flashbacks.”* (Patient 2).

This account from Patient 2 demonstrates the dual impact of self-tracking. While it increased awareness and emotional engagement with his traumatic experiences, it also amplified his distress. The process of repeatedly pressing the button, much like the recurrent nature of traumatic flashbacks, escalated his anxiety and highlighted the constant struggle or ‘war’ between him and his traumatic memories, represented by the instrument.

Patient 2 vividly shared the emotional burden, stating:

*“(…) it might help others, but for me it only gave a negative result”* (Patient 2).

These observations illuminate the significant emotional impact of self-tracking, particularly when tracking distressing phenomena (39). For Patient 2, the OBT inadvertently served as a stimulus related to his traumatic memories, leading him to focus on these distressing experiences. In effect, the act of self-tracking forced him to confront these trauma-related stimuli, which he had typically avoided before treatment started. Interestingly, Therapist 2 acknowledged this unintended exposure during the final therapy session as documented in the logbook entries. However, there was a noticeable absence of this dynamic in earlier entries, suggesting a possible underestimation or oversight of the emotional toll tied to the self-tracking process. Furthermore, the negative impact on Patient 2 could also be linked to his attempt to conceal his treatment and OBT usage from his family and social environment. This concealment could have amplified his feelings of vulnerability, underscoring the multifaceted implications of self-tracking in a therapeutic context.

#### Theme 2: OBT as a sign of treatment

The communicative aspect of the OBT was also highlighted in patients’ daily lives, but unlike in the therapy sessions, wearing the OBT was perceived as a signal to others that they were undergoing treatment. The public visibility of the OBT introduced a complex dynamic, creating a continuum of experiences from enabling supportive dialogues to engendering stress and stigma. On one end of the continuum, the OBT acted as a facilitator for conversation and understanding within the family. For instance, Patient 1 found that the device served as a point of discussion about his mental health with his family members. The very presence of the OBT in his everyday life led to a heightened awareness of his mental health struggles among his family, notably his wife and youngest son. His wife, in particular, was supportive and actively encouraged him to use the instrument effectively:

*“Sometimes I forget that when I’m in pain, I should press the button. My wife reminds me and says to me that I have to press the button when I’m in pain.”* (Patient 1).

This illustrates how the OBT can foster communication, and awareness, and inspire familial support for the patient’s mental health journey, transforming the treatment into a collective, rather than isolated, endeavor. However, it also highlights the complex interplay between the OBT and the social context in which the patient is situated.

At the opposite end of the continuum, the visibility of the OBT was a source of stigma and distress. This was the case for Patient 2, who chose to conceal his treatment from his family out of fear of judgment and stigmatization:

*“I kept it hidden because I knew how she [his wife] would react. Once, she noticed there was something in my pocket and asked what it was. I told her it was a watch…”* (Patient 2).

In this context, the OBT became a symbol of a stigmatized treatment, turning its daily use into a source of stress rather than a therapeutic process. The act of hiding the instrument may have exacerbated his stress levels, thereby potentially hindering the therapeutic process. This dichotomy underscores the complex interplay between the OBT, the cultural framework, and the social context in which the patient is embedded. While the OBT can act as a catalyst for supportive dialogue, it can simultaneously evoke a sense of stigma, possibly especially in individuals from a minority ethnic group where the stigma of mental illness remains a sensitive topic (40). Thus, culture-specific factors and societal perceptions of mental health within each patient’s environment also affected the treatment experience including using the OBT.

#### Theme 3: following the doctor’s order

The third theme provides insight into patients’ perception of the OBT as a prescribed tool integral to their treatment. It was evident from the interviews that both patients considered the OBT a non-negotiable part of their treatment, an attitude reflected in their consistent use of the instrument, which signifies a commitment to the therapy. For instance, Patient 1, acknowledged that despite the discomfort of pressing the button during instances of pain, he remained compliant with the treatment:

*“Even though it is sometimes unpleasant, I have to press the button when I am in pain. Because I promised to use the treatment.”* (Patient 1).

The OBT not only served as a tangible bridge between therapy sessions and daily life experiences, but also represented an implicit treatment contract, committing both patient and therapist to the therapeutic process. This commitment, however, revealed an intriguing discrepancy in the understanding of treatment engagement. Despite the therapist expressing concerns in the logbook entries about the patient’s level of active participation in therapy, the patient’s commitment to the OBT suggested a high level of participation. While the therapist expected the patient to draw personal insights into his mental health issues, following the personal science perspective (21), the patient seemed more focused on the communicative aspect of both the OBT and its generated data:

*“He has no judgment about whether he can benefit from it or not. Because he doesn’t know. He only uses it to show when he is in pain. Nothing else.”* (The interpreter on behalf of Patient 1).

This misalignment between the therapist and patient’s understanding of self-tracking and overarching treatment goals echoes the discrepancy noted in Theme 2 in the context of therapy sessions. Patient 1’s statement further elucidates this divergence:

*“I just followed what [his therapist] told me. I had to press when there were symptoms or pain. I have no judgment on whether I can benefit from it or not.”* (Patient 1).

As illustrated in Therapist 2’s final logbook entry, Patient 2 continued to use the OBT despite expressing a desire to discontinue its use, further emphasizing the perception of the tool as an integral, non-negotiable part of the treatment. This raises questions about the factors at play in the therapy context possibly reflecting a complex interplay between patient compliance, perceived power dynamics, and the communication of discomfort or uncertainty in the therapeutic relationship. In response to Patient 2 expressing his desire to discontinue the use of the OBT, Therapist 2 reflected on potential power dynamics:

*“My immediate reaction was how long have you felt this way? How long did it take for you to gather the courage to tell me?”* (Therapist 2).

This reaction from the therapist signals an awareness of the power dynamics inherent in the therapeutic relationship. The therapist recognizes the courage it took for the patient to express his desire to discontinue the OBT, suggesting that the therapist is attentive to the potential power imbalances and their impact on the therapeutic process to avoid an overly compliant patient at risk of violating personal boundaries.

## Discussion

This article explores the integration of a self-tracking instrument, the OBT, into psychotherapy with refugees diagnosed with CPTSD, examining its influence on patient engagement and how it becomes a part of psychotherapeutic treatment. Anticipating lower levels of engagement due to the known challenges of active data collection with wearable devices (41), our findings illustrate how a single-purpose self-tracking instrument can play a pivotal role in reaching a high patient engagement and personalizing the treatment approach.

### Enhancing engagement through an active patient role

In response to RQ1, our feasibility pilot study demonstrated a high level of patient engagement in psychotherapeutic treatment extending beyond therapy sessions and integrating into patients’ daily lives. This enhanced engagement is particularly evident in the robust use of the OBT. It’s noteworthy to consider this finding in contrast with other digital mental health applications that similarly propose self-monitoring yet often report low user engagement (42). For instance, in an EMA feasibility study by Moitra and colleagues (43), participants, recently discharged from hospital care for psychotic disorders, completed 30% of EMA surveys over a month-long period. This limited engagement persisted despite weekly technology assistance provided by the study staff. This gap in engagement underscores a crucial point of differentiation between our study and many other digital mental health studies. Unlike these studies, this study emphasizes the collaborative identification of a subjectively defined relevant target phenomenon by the patient and therapist, within the framework of the self-tracking assisted treatment concept. We postulate that this collaborative process enhances the perceived relevance of the OBT for the patient, fostering their engagement with the tool. This dynamic resonates with the research indicating that mobile apps, when utilized in a coached condition or as an adjunct to treatment, result in a higher degree of patient engagement (44). This patient-centric model is particularly impactful within the scope of interpreter-mediated psychotherapy (20).

These findings contribute to the discourse on patient engagement and digital health tools by underscoring the potential of minimalist, user-friendly self-tracking instruments, like the OBT, in eliciting high user engagement and as such can serve as powerful tools for gathering dense, patient-specific data over extended periods (35, 41).

Engaging patients with tasks between sessions, such as homework, is a key strategy for extending treatment beyond the therapy sessions (45). While the ability of traumatized refugees to engage in homework exercises has been questioned (17), research suggests a correlation between high homework compliance during CBT for refugees and improved mental health symptoms and social functioning (46). Typically, therapists primarily define homework tasks in psychotherapeutic treatment, potentially relegating patients to a passive role. The introduction of the OBT as homework, however, creates a paradigm shift: It empowers patients to define their target phenomena and to collaboratively analyze the collected data with their therapist (22). This shift transforms patients’ homes and daily routines into healthcare data collection sites, thereby creating a ‘digitally engaged’ patient role (39). Such autonomy and personal relevance could account for the high engagement with the OBT seen in our study.

The active role patients adopted in therapy, as demonstrated by their engagement with the OBT, allows for deeper insight into their lived experiences, echoing prior research emphasizing the significance of active patient participation in integrating healthcare technologies into psychotherapeutic processes (47–49). However, daily self-tracking can have drawbacks, as demonstrated by one participant in our study who experienced elevated anxiety levels while self-tracking, an observation that aligns with other studies using self-tracking instruments (35, 50). This suggests the need for careful and mindful integration of such technologies into treatment (51).

The significant role of digital mental health interventions in patients’ daily lives was evident in our research, reflecting Borghouts et al.’s (25) systematic review. In this context, the OBT’s integration into daily routines offers therapeutic possibilities for refugees, indicating its feasibility. However, the usage and perception of the OBT varied between therapy sessions and daily life, indicating the importance of contextual factors in determining the effectiveness of such technologies (52). In our study, the therapists predominantly addressed the OBT usage during therapy sessions and there was no effort to reach out to patients between sessions to encourage them to use the OBT. This finding suggests the necessity for further research into strategies for better integrating digital mental health interventions into refugees’ daily routines.

Consequently, while this feasibility pilot study posits that self-tracking instruments such as the OBT have potential in the treatment of refugee populations diagnosed with CPTSD, particularly in augmenting their active engagement and autonomy in the therapy process, it also highlights some challenges. Therapists must remain mindful of how patients interpret data indicative of deteriorating mental health (39, 53), underscoring the importance of professional guidance in ensuring self-tracking technologies contribute positively to therapeutic goals, rather than inadvertently hampering them. This insight highlights the importance of personalization in treatment strategies, particularly in enhancing homework compliance and overall patient engagement. Our study provides empirical support for the feasibility of integrating the OBT within the context of intercultural psychotherapy. Moreover, the promising findings from our study underscore the importance of simplicity and user-friendliness in designing mental health interventions. These elements not only enhance patient engagement with therapeutic tasks such as homework but also affirm their active role in the therapeutic process, thereby facilitating a more personalized therapeutic approach. This approach, sensitive to the unique circumstances of each patient, appears to be paramount in fulfilling the potential of such self-tracking instruments in the treatment of CPTSD among refugees.

### Using the OBT to personalize treatment

In line with the discourse of the digitally engaged patient (39), the self-tracking instrument used in this feasibility pilot study, the OBT, transcended its initial purpose of data collection. It promoted active patient participation and facilitated the development of a personalized therapeutic approach.

Addressing RQ2 on how personalized self-tracking data become a part of the psychotherapeutic process, this feasibility pilot study revealed from the patients’ perspective that the OBT was not merely a neutral tool, but it played a pivotal role in their therapy experience. The OBT held a dual function: (1) The OBT served as a tool for self-monitoring and (2) as a tangible representation of their treatment contract with their therapist.

The OBT allowed patients to engage in what can be termed as participatory self-surveillance, as they gathered data on their selected target phenomena in their day-to-day environments (22, 39). The OBT made the often elusive target phenomenon more concrete, providing patients with a medium to express their experiences through data (54). As an embodiment of the therapeutic contract, the OBT fostered a more intimate connection between patient and therapist, echoing previous findings that digital health technologies often weave emotional and social ties with healthcare providers (39, 50).

Our study supports the transformative potential of using self-tracking instruments and personalized data in psychotherapy. The study also highlights that tracking can reduce as well as amplify target phenomena (52, 55). This effect underscores the necessity for therapists to pay attention to how technology can influence a patient’s therapeutic journey. Likewise, it is crucial to recognize that transformative experiences can differ substantially among patients, influenced by factors such as cultural expectations, personal experiences, and therapeutic strategies (56). The OBT also served as a bridge between patients’ daily lives and therapy sessions, thereby enhancing the therapeutic process by increasing the treatment dosage (45). For instance, Patient 1 used the OBT to facilitate mental health discussions with family members and his therapist. This finding aligns with recent research that identified social connectedness as a facilitator of engagement in technology-mediated interventions (25, 57). Patient 2, however, experienced an unintended exposure situation, demonstrating the instrument’s variable impacts on individual psychotherapeutic processes. Consequently, the integration of self-tracking wearables into psychotherapy should focus not only on data collection but also on its interpretation and application in improving therapeutic outcomes (58). As discussed by Lupton (22) an ethical dilemma emerges regarding differing perspectives on the overall purpose of self-tracking, including the notion of moral responsibilities of self-improvement following insight and awareness. The prevalent notion that endorses self-monitoring as a hallmark of personal growth and management may inadvertently present challenges for individuals who do not share the same outlook toward self-tracking (50). As such, careful consideration is needed when incorporating self-tracking into psychotherapy, mindful of potential discrepancies in its perception and usage.

From the therapists’ perspectives, it became evident that integrating the OBT into treatment required modifications to traditional therapeutic approaches, thereby posing certain challenges. The main challenges revolved around integrating the data into a personalized, data-driven therapeutic approach, underlining the intricacies of tailoring treatment, especially within refugee populations. Our findings highlight the need for education and supervision to unfold the potential of a personalized, data-driven treatment approach using the OBT.

Furthermore, the emerging trend of patients engaging digitally to gain insights signals a transformative shift in traditional psychotherapy, with technology like the OBT structuring the treatment (39). As cultural expectations can significantly influence therapeutic experiences (56), the findings in the theme “Following the Doctor’s Order” emphasize the need for careful appraisal of technology’s suitability for individual patients (59).

However, our feasibility pilot study revealed an additional layer of complexity. The virtual presence of an interpreter, while necessary in some circumstances, can potentially exacerbate the inherent challenges of establishing shared understanding in intercultural psychotherapy. Successful integration of self-tracking wearable instruments in such a context necessitates addressing and overcoming these challenges.

## Limitations

While this feasibility pilot study breaks new ground by integrating the use of self-tracking data into psychotherapy with refugees, it is important to acknowledge its limitations. Due to the nature of the study being a feasibility pilot study, the study only included two patients and two therapists. Logbook entries were sparse, creating gaps in our understanding of the therapeutic process and the specific use of data within individual sessions. This suggests a need to systematize the use of logbooks in the main case series. We also observed that the virtual presence of an interpreter introduced an additional layer of complexity unrelated to the use of the OBT. Future research should consider exploring alternative interpretation methods that do not add complexity to the therapeutic process. Nevertheless, despite these limitations, our study offers valuable insights into the potential benefits and challenges of integrating self-tracking wearables in psychotherapy, thereby advocating for more extensive research in this area.

## Conclusion

This feasibility pilot study sought to explore the feasibility of integrating the OBT into psychotherapy with refugees diagnosed with CPTSD. Our findings suggest the feasibility of employing such single-purpose self-tracking technologies within the context of intercultural psychotherapy, thereby encouraging high patient engagement and highlighting potential advancements within this innovative treatment paradigm. By catalyzing substantial patient engagement and enabling personalization of the therapeutic process, these tools may significantly impact the future of psychotherapeutic treatment strategies for refugees with CPTSD. However, the incorporation of the OBT into psychotherapeutic treatment necessitates a more nuanced approach than a simple one-size-fits-all model. It calls for an adaptive treatment concept with well-defined procedures for personalization, designed to adapt to the evolving goals, context, and psychological states of the patients. Consequently, additional research is warranted to enhance our understanding of these dynamics and refine the utilization of self-tracking instruments in intercultural psychotherapy. This exploration should include a focus on the potential risks of symptom exacerbation and the necessity for tailoring treatment models to individual patients.

Key findings from our study hold implications for both practice and research. Therapists can use OBT data in conjunction with patients’ qualitative tracking experiences to gain a nuanced insight into the target phenomenon and identify potential patterns. Such integration can enrich the therapeutic experience by consolidating the connection between the patient’s daily life and the therapeutic process. Nonetheless, therapists must maintain vigilance regarding possible risks of symptom exacerbation, managing these risks with meticulous care. They should also be aware of potential ethical dilemmas tied to an uncritical idealization of self-tracking as a pathway to self-management and personal growth. Furthermore, in intercultural psychotherapy contexts, therapists need to consider the potential stigma associated with using the OBT within the patient’s familial and cultural framework (56). Given these promising results, more comprehensive research is required to further investigate and develop the application of single-purpose self-tracking wearables in psychotherapy.

## Data Availability

The raw data supporting the conclusions of this article will be made available by the authors, without undue reservation.

## Footnotes

1 Roberts NP, Cloitre M, Bisson J, Brewin CR. International trauma interview (ITI) for ICD-11 PTSD and complex PTSD. (2018). Unpublished measure.
